# Supplementary material for: Zonarol Protected Liver from Methionine- and Choline-Deficient Diet-Induced Nonalcoholic Fatty Liver Disease in a Mouse Model
Source: Nutrients. 2021 Sep 29;13(10):3455. doi: 10.3390/nu13103455 (PMC8537643; doi:10.3390/nu13103455)
Supplement: Supplementary file 1 [file nutrients-13-03455-s001.zip › nutrients-1370895-supplementary.pdf]

Table S1. Antibody used in this study.

Antibody	Manufacturer	Catalog No.
β-Actin	Cell Signaling Technology	#4970
SREBP-1/2	Bioss	bs-1402R
PPARγ	Abcam	ab59256
IL-1β	Cell Signaling Technology	#12242
TNF-α	Abcam	ab66579
NF-κB	Cell Signaling Technology	#8242
Nrf2	Gene Tex	GTX103322
HO-1	Cell Signaling Technology	#43966
NQO1	Santa Cruz	sc-32793
keap1	Abcam	ab139729
Akt	Cell Signaling Technology	#9272
phosphor-Akt	Cell Signaling Technology	#9271
PI3K	Cell Signaling Technology	#4257
phosphor-PI3K	Cell Signaling Technology	#4228
Anti-rabbit IgG	Cell Signaling Technology	#7074
Anti-mouse IgG	Cell Signaling Technology	#7076
Anti-Mouse/Human Mac-2 Antibody	Cedarlane	CL8942AP
anti-human α-smooth muscle actin antibody	Dako Cytomation	M0851
8-OHdG	Japan Institute for the Control of Aging	MOG-100P
